# Some Recent Surgical Work*Read at the last annual meeting of the Georgia Medical Association.

**Published:** 1898-08

**Authors:** Hunter P. Cooper

**Affiliations:** Atlanta, Ga., Professor Anatomy and Clinical Surgery, Atlanta College of Physicians and Surgeons


ATLANTA

### Medical and Surgical Journal.

VOL. XV. AUGUST, 1898. NO. 6.

L. B. GRANDY, M.D., DUNBAR ROY, M.D., EDITORS.

M. B. HUTCHINS, M.D., BUSINESS MANAGER.

### ORIGINAL COMMUNICATIONS.

### SOME RECENT SURGICAL WORK.*

1. NEPHRO-LITHOTOMY. 2. HIP-JOINT AMPUTATION FOR SARCOMA. 3. CRANIECTOMY. 4. IMPACTED CALCULUS.

BY HUNTER P. COOPER, M.D., ATLANTA, GA.,

Professor Anatomy and Clinical Surgery, Atlanta College of Physicians and Surgeons.

The cases which I wish to report have been operated on either in my hospital service or in the Elkin-Cooper Sanatorium, and are reported because they are deemed of sufficient interest to bring to the attention of the members of this Association.

The first case is one of nephro-lithotomy. In brief, the history of the case is as follows:

Dr. M., aged thirty-eight, physician, had suffered with attacks of sharp pain in the right side throughout his life. These attacks varied very greatly in frequency and severity. The pain was always referred to the right side, between the crest of the ilium and the twelfth rib. During such attacks the urine always became

*Read at the last annual meeting of the Georgia Medical Association.

bloody. In the last ten years pains have been much more acute, although between attacks he still remained free from pain. Two years ago he noticed pus in the urine. This soon became constant, and was present in large quantities. In November last he went to New York and was operated on by Dr. Wyeth. He entered the kidney on its posterior surface and let out a large quantity of pus accumulated in the pelvis of the organ. Thorough drainage was established and the patient soon returned to his home in South Georgia. His pains, however, were never relieved to any extent by the operation, and when he was admitted to our sanatorium on January 19th of this year he was a pitiable object. His suffering was almost constant. An exploration of the kidney was advised, with the idea of removing the organ, if possible; and if not possible, to establish a freer drainage of the abscess cavity. On January 21st he was anesthetized and a crucial incision made in the lumbar region, Dr. Elkin being present and rendering me valuable aid and counsel. The vertical limb of the incision extended from the twelfth rib to the crest of the ilium, the horizontal limb from the transverse vertebral processes forward about nine inches. This extensive incision was necessary on account of the patient being a very stout man. The incision was deepened until I came in contact with the kidney. A small opening was found on its posterior surface, which I cut, letting out a considerable amount of pus. The finger was then introduced into the enlarged pelvis of the kidney, and on careful examination a calculus was discovered lodged in the substance of the kidney and presenting in the cavity. This was shelled out, and found to be a large conical calculus nearly an inch in its longest diameter, and having somewhat the shape of an Indian arrow-head. No other calculus being discovered, and the kidney appearing sound enough to remain behind, it was decided to leave it. The future history of the case was very satisfactory. The highest temperature recorded was $100\frac{3}{5}$. There still remains a fistulous opening in the lumbar region, through which urine and some pus are passed.

CASE 2. *Amputation at the Hip-joint for Osteo-sarcoma of the Femur.*—Especial interest in this case is due to the fact of a sarcoma rapidly following a traumatism.

Mr. R., aged thirty, was admitted to the Elkin-Cooper Sanatorium December 10, 1897. On November 14th his left leg was crushed by a wheel of a railroad train, necessitating amputation above the knee. The operation was done in the town where the accident occurred. Infection followed the operation, and septicemia resulted. When he was brought to the sanatorium his temperature ranged from 102 to 104, pulse from 120 to 136, and he was very weak and emaciated. The stump was unhealed, the end of the bone protruding an inch beyond the flaps. The abscess extended from the very end of the stump to within three inches of Poupart's ligament. He was anesthetized and the abscess opened fully by an eight-inch incision on December 13th. A few days later the end of the bone was removed and the granulations curetted. His general condition improved rapidly for a while, but it was soon noticed that large knob-like masses were rapidly forming around the end of the bone. These grew with great rapidity. One of these was cut off and examined microscopically by Dr. Bourns, professor of pathology in the Southern Medical College. He reported it to be a large-celled sarcoma. Amputation at the hip-joint was thereupon advised, and consented to. It was done January 18th, Wyeth's bloodless method being used. Two large drainage-tubes were inserted, and the patient recovered without any difficulty. The highest temperature recorded subsequent to the operation was $100\frac{2}{5}$, the temperature ranging most of the time between $98\frac{3}{5}$ ane $99\frac{1}{2}$. He was discharged on February 23d, the wound being entirely healed, except where the drainage-tubes were brought out. I have seen him very recently; he is in perfect health and has gained very greatly in weight. Throughout the operation and treatment of the case I was assisted by my associate, Dr. Elkin.

CASE 3. *Craniectomy for the Relief of Imbecility.*—H. W., white, seven years old, was admitted to my service in the Grady Hospital on the 7th of December, with the following family and personal history:

Mother has three living children, four dead, and has had two miscarriages. Two of the living children are well and healthy. The four died from the following causes: First, premature labor, seven months; second, malnutrition, aged five months; third, malnutrition, aged two months; fourth, cause unknown, aged six months. They were all bright mentally, but never grew physically. The mother has a second-cousin who is an imbecile.

*Past History.*—When patient was born mother was in labor forty-eight hours. Labor was severe. Baby weighed twelve pounds. The child's head showed signs of bruises, and there existed for several days a large area of extravasated blood over the occiput and one side of the head. After nine days the head had regained its normal shape, and was said to be a very finely-shaped head. On the ninth day and seven succeeding days patient had convulsions, one to three a day; these were accompanied by high fever. During these convulsions the frontal suture enlarged. Following this, the sagittal and lambdoid also opened, and at the fontanelles the membranes bulged out. The spasms were clonic, affecting all the muscles of the body, but seemed to be worse on the left side. Child very stupid for next three weeks. After this, child's head gradually returned to normal shape; child brightened and nursed; became well nourished; walked at sixteen months. When sitting, head had a tendency to fall forward on chest. When the head was reduced in size the bones of the right side overlapped those of the left. The two troubles just mentioned were all that bothered the patient at eighteen months. At this time she had a chill, followed by another in a few hours. The last one was followed by a congestion of the brain. Patient had severe spasms and loss of consciousness, lasting twenty-four hours. Typhoid fever followed, as diagnosed by family physician, lasting seven weeks. Afterwards patient did not walk for one year, and did not talk for two. She was a bright, intelligent child at eighteen months, when last trouble came on. Development has been slow since then.

*Present History.*—Patient is an imbecile. Sentences consist of only two or three words. Makes her wants known without much trouble. Paresis and atrophy on left side; left leg drags when walking. Head is very small and very narrow from side to side, especially in frontal and parietal regions. Child is self-willed, irritable, nervous, and suffers from insomnia. Subject to fits of uncontrollable rage and utterly devoid of reason.

*On Admission.*—Tongue fairly clean. Appetite fair. Bowels regular. Pulse 100. Temperature 98.

*Diagnosis.*—Imbecility. Intra-cranial space too small to permit brain development.

*Operation.*—Craniectomy advised. Mother consented. On the 9th of December patient was prepared in the usual manner, and on the 10th I made the operation, making an incision beginning at hair margin in front, about half an inch to right of sagittal suture, and carrying it back to the lambdoid suture. Incision slightly curved. The flap was turned back; also the pericranium. I next trephined the skull, and with the cutting forceps removed the bone forward for two and one-half inches and backward for about two inches, and then to the side for about one and one-half inches. The space between the two edges of the bone is about an inch wide. All hemorrhage was stopped, there being very little. Wound was slightly packed with iodoform gauze and sutured. Usual dressing applied. Dressed first on the third day; wound in good condition. Stitches removed on the tenth day; union perfect, except space left for drainage. Patient now made an uninterrupted recovery. Bowels kept open with oil. Action caused on the fourth day after operation. Mother noticed some change for the better in the child at once. Patient was discharged on the 28th of December. Great improvement in the mental faculties followed the operation. She became obedient, had no more fits of rage, talked much better and seemed like a different creature.

By my advice patient returned in May of this year to have a similar operation performed on the opposite side of the head. It was done in the same manner as before, and healing took place by first intention throughout. Patient's mental condition has been wonderfully improved by the two operations, and I have no doubt will continue to improve for some time to come.

CASE 4. *Stone Impacted in Membranous Urethra.*—W. R. J.; child; four years old. Was admitted to my service in Grady Hospital on the 14th of December, 1897, with the following family and personal history.

*Past History.*—Seven months ago it was noticed that the patient became very fretful when he wanted to urinate, and seemed to suffer very much during the act. It was noticed that the urine came very slowly, and in a very small stream. He gradually grew worse, urinated more frequently, and had much pain each time. Stream grew smaller, and for the last four months the urine has just been dribbling. Passed no calculi or blood.

*Present Condition.*—Patient has suffered from retention of urine for thirty-six hours. A little urine was passed involuntarily on the train—very little, however. The bladder reached to the umbilicus, and as no instrument could be passed into the bladder, patient was aspirated and a large quantity drawn off. With the steel instrument a calculus could be felt in the membranous urethra, very near the prostate gland.

*Diagnosis.*—Impacted stone in deep urethra. Operation advised and consented to by father.

*Operation.*—On the morning of December 14th, just after patient came in, he was prepared for operation, anesthetized and placed on the table. I made a suprapubic cystotomy, and then a perineal section, the stone, which was the size of a small bean, being removed by the latter operation. There were no calculi in the bladder. A small catheter was passed into the bladder through the perineal wound and fastened for drainage. I irrigated the bladder with a boric solution, then caught up the bladder and fastened it on either side with a suture to the skin, put in a strip of iodoform gauze, and then dressed both wounds in the usual manner.

*After Treatment.*—The bladder was irrigated daily with boric solution and fresh dressings applied each time. On the fifth and sixth days after the operation temperature went up, but came down to normal immediately. Bowels moved on third day. Sutures and drainage-tube removed on the seventh day. Wound in good condition. Patient made a good recovery and was discharged on the 11th day of January, 1898.

---

ACCORDING to the *Medical Summary* (*Western Medical Review*, June 15th), the injection of a glass syringeful of lemon juice into the nose, after it has been cleaned of clots, will stop bleeding after everything else has failed.

